# Gender Differences in Faculty Rank and Leadership Positions Among Physician Biochemistry Faculty in North America: A Retrospective, Cross-Sectional Study

**DOI:** 10.7759/cureus.20731

**Published:** 2021-12-27

**Authors:** Muhammad Haaris Tiwana, Irina Sverdlichenko, Lisa Xuan, Sabeena Jalal, Sabeen Tiwana, Fajr Khawaja, Faisal Khosa

**Affiliations:** 1 Dentistry, Lahore Medical and Dental College, Lahore, PAK; 2 Epidemiology and Biostatistics, University of Western Ontario, Vancouver, CAN; 3 Medicine, University of Toronto, Toronto, CAN; 4 Radiology, University of Ottawa, Ottawa, CAN; 5 Radiology, Vancouver General Hospital, Vancouver, CAN; 6 Dentistry, University of British Columbia, Vancouver, CAN; 7 Sciences, Wellington College, Crowthorne, GBR

**Keywords:** retrospective research, medical biochemistry, research productivity, academic rank, gender-based differences

## Abstract

Purpose

This study sought to assess gender differences among physician faculty in medical biochemistry and genetics programs in North America. It compared the distribution of academic and leadership ranks, years of active research, number of citations and publications, and Hirsch-index (*h-*index) by gender. Variable associations with the *h*-index were assessed.

Method

This was a cross-sectional retrospective study for which data was collected from June 2019 to October 2019 on academic and administrative physician faculty members for medical biochemistry and genetics programs. The website of Fellowship and Residency Electronic Interactive Database, Canadian Resident Matching Service website, and the medical biochemistry profile from the Canadian Medical Association to identify relevant programs and SCOPUS was used to gather faculty data.

Results

The analyses included 147 faculty members. More male faculty held higher academic rank positions and first-in-command leadership positions than female faculty. Men had more median years of active research, citation numbers, publication numbers, and *h*-index than women across all academic ranks. Upon performing multivariable linear regression, female faculty showed 0.39 times the odds of having a higher *h*-index than male faculty, keeping all other variables constant (p<0.01).

Conclusions

In our study, it was shown that male physician faculty surveyed had higher performance than female faculty in academic rank and research productivity. Certain barriers may be contributing factors, including lack of mentorship or flexible institutional policies, women choosing clinical educator tracks, or gender bias. Considering the low retention rates of women in academic research, there is a need to address barriers in order to achieve gender parity.

## Introduction

Over the past decades, efforts have been directed towards the advancement of women in science, technology, engineering, and mathematics (STEM). While some STEM academic disciplines like mathematics and statistics still lag behind in terms of equal gender representation, academic disciplines like the life sciences have shown encouraging results. For instance, statistics on earned degrees in 2012 showed that women were awarded 59% of bachelor’s degrees in biological/biomedical sciences and 54% of doctorate degrees in this discipline [1].

Despite the increasing representation in undergraduate and doctoral programs, gender disparities continue to persist in academic disciplines [2, 3]. One meta-analysis showed that men are more successful than women in gaining faculty and research positions as well as nominations for evaluation committees and grants [3]. While the factors leading to these disparities are still under debate, one claim is that differences in scientific output play a role [3]. The scientific productivity of a researcher is typically measured by their h-index, which depends on both the number of a scientist’s publications, as well as the citations of these publications [4]. The h-index is used to determine the past and predict the future research productivity of a scientist [4, 5]. For this reason, it is often looked upon favourably when determining academic appointments and allocating research resources [5]. 

Studies have shown that women researchers tend to have lower scientific productivity than men [2]. A cross-disciplinary bibliometrics analysis revealed that women were responsible for fewer than 30% of fractionalized authorships worldwide [6]. Furthermore, manuscripts written by women holding prominent author positions attracted fewer citations than those written by men holding similar positions [7]. Thus, women’s comparatively lower research productivity and citations may influence indices used to determine potential promotions.

Factors that may influence women’s productivity in academia include years they are actively pursuing research endeavours and gender bias. Women more often than men interrupt their careers to have children and start a family, taking maternity leave at a time when they could be building their publication record [8]. Furthermore, gender bias in the scientific community continues to persist. One study found that gender disparities were visible in committee evaluations of the applicants, including prioritization of applicants’ quality as a researcher but not the quality of their research proposals, and in language use in instructions and evaluation sheets.

The underrepresentation of women in faculty leadership can have negative consequences including same-sex mentors and role models [9]. The purpose of this study was to understand the prevalence of gender differences in the medical biochemistry and genetics discipline and their impact on research productivity. Specifically, we looked at gender distribution of medical biochemistry and genetics faculty by academic ranks (i.e., assistant professor, associate professor, and professor) and leadership ranks (i.e., first-in-command and second-in-command), differences in years of active research, median publications, and citations. Furthermore, the h-index of scientists and medical biochemistry and genetics faculty by gender, across different academic ranks were assessed, along with factors affecting the same.

## Materials and methods

This was a record-based cross-sectional study for which the authors collected data from June 2019 to October 2019 using previously validated methodology [10]. Informed consent or institutional review board approval was not required as all data used for the study were publicly available. We gathered data for academic and administrative faculty members for all medical biochemistry and genetics departments across all programs in the United States (US) and Canada using various resources. We used the search term "medical biochemical genetics" on the Website of Fellowship and Residency Electronic Interactive Database (FREIDA) online. This provided us with a list of 16 Accreditation Council for Graduate Medical Education (ACGME) - accredited Medical Biochemistry and Genetics programs of the American Medical Association (AMA). For the medical biochemistry and genetics programs in Canada, we referred to the Canadian Resident Matching Service (CaRMS) website, which provided us with a total of one program for medical biochemistry and seven programs for medical genetics. Additionally, the medical biochemistry profile from the Canadian Medical Association (CMA) allowed us to identify five additional schools and two additional medical biochemistry programs in Canada. Using the stated websites, we compiled a list of medical biochemistry and genetics programs that offered the faculty listings on their respective websites. We excluded programs (n=9) if they either did not have the faculty listing available or did not report administrative or faculty ranking. After excluding these programs, we obtained 14 programs in the US and eight programs in Canada.

We reviewed the websites of the 22 selected programs for their faculty listings, which we extracted into our database for analysis. Inclusion criteria were full-time faculty members with the academic ranking of professor, associate professor or assistant professor with a Doctor of Medicine (MD) or Doctor of Osteopathic Medicine (DO) degree and a listing on the university website. We also included faculty with departmental leadership roles such as chair, vice-chair, director, associate/assistant director, department head, or chief. Furthermore, the first-in-command and second-in-command were defined using the respective program's leadership hierarchal structure i.e., "first-in-command" was the current senior-most leadership position i.e., first on command included Department Chair or Head of Department and second in command included Vice-Chair and Program Director. We excluded adjunct and retired faculty from our search. Faculty whose gender could not be identified based on bibliographic information were excluded. Lastly, we used Elsevier’s SCOPUS to gather data pertaining to article publications, h-index, citations, and years of active research of each faculty member. SCOPUS was chosen because it is a reliable tool to measure h-index [11]. It has been found to capture more citations than Web of Science as well as to capture citations of higher scientific impact (excluding such citations as theses, conference papers and unpublished works) as compared to Google Scholar [12].

Statistical analysis

We tested the data for normality and then performed log transformation for the continuous variables of h-index, number of citations and number of publications, which were skewed in distribution. At the univariate level, we applied simple linear regression for the independent variables of gender, academic rank, leadership rank, and years of active research. We regressed each variable individually with h-index. Since the data did not follow normal distribution after running a Kolmogorov Smirnoff test, we ran non-parametric tests and checked for the median and ranges. Upon univariate regression, our team identified gender, publications, citations, academic rank, and leadership rank to be significant, while years of active research were insignificant. The significant variables were selected for inclusion into multivariable linear regression analysis. We then checked multi-collinearity between independent variables using a correlation coefficient. Cramer’s V test was used for one nominal variable and one ordinal variable, and Spearman’s test was used for one continuous variable and one ordinal variable. We treated a correlation of 0.8 as the presence of multi-collinearity. Finally, we created interaction terms between each of the main effects in the model. SPSS Statistical Software Version 23 (IBM Corp., Armonk, NY) was used for running statistical analysis.

## Results

Our search identified 173 faculty members who met inclusion criteria for our study. Information on gender was available for 148 faculty members, 147 of whom also had their academic rank listed (Table 1). The final sample consisted of 58 (39.46%) female faculty and 89 (60.54%) male faculty.

**Table 1:** Male and female proportions of academic ranks and leadership positions n refers to 'number of' Values in parentheses are percentages.

		Males	Females
Academic ranks (n = 147)		n = 89 (60.54%)	n = 58 (39.46%)
	Assistant Professors	20 (22.47%)	22 (37.93%)
	Associate Professors	28 (31.46%)	12 (20.69%)
	Professors	41 (46.07%)	24 (41.38%)
Leadership positions (n = 48)		n = 33 (68.75%)	n = 15 (31.25%)
	First-in-command	31 (93.94%)	12 (80%)
	Second-in-command	2 (6.06%)	3 (20%)

**Figure 1:**
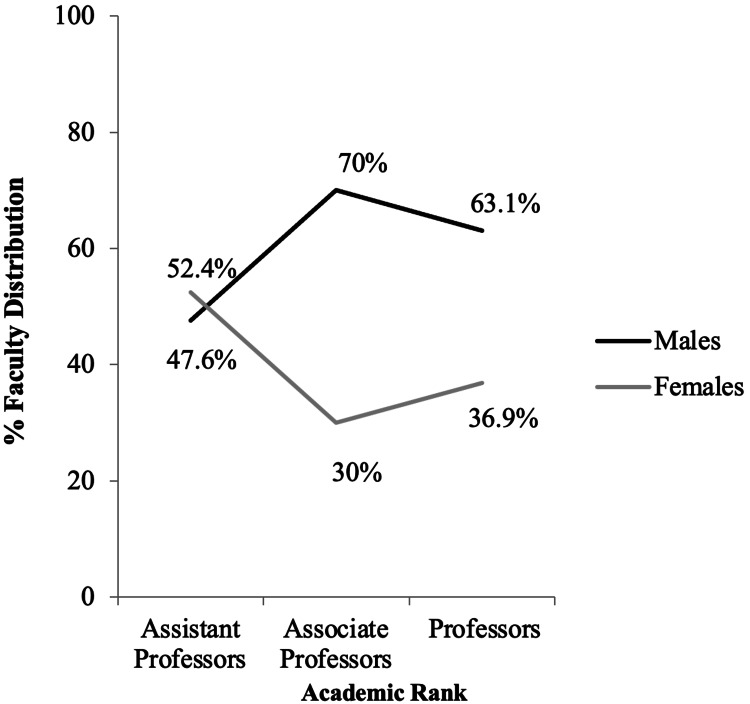
Percent distribution by gender of 147 faculty members across academic ranks in medical biochemistry and genetics programs in North America in 2019.

Among faculty members, 42 (28.57%) were assistant professors, 40 (27.21%) were associate professors and 65 (44.22%) were professors. There were 20 (47.6%) male assistant professors, 28 (70%) male associate professors and 41 (63.1%) male professors (Figure 1).

**Figure 2:**
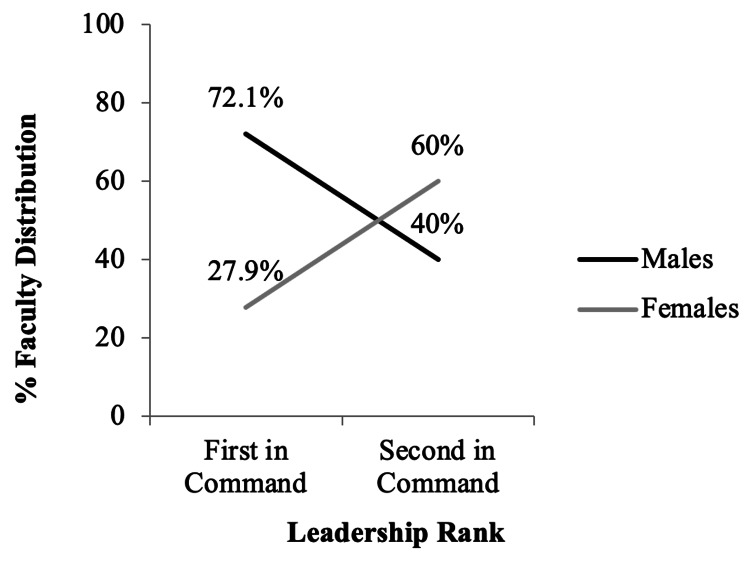
Percent distribution by gender of 48 faculty members across leadership ranks in medical biochemistry and genetics programs in North America in 2019.

Our data also revealed that 48 of the faculty members held leadership positions. Among those holding leadership positions, 43 (89.6%) faculty members were first-in-command and five (10.4%) were second-in-command. For those first-in-command, 31 (72.1%) were males, and for those second-in-command, two (40%) were males (Figure 2).

**Figure 3:**
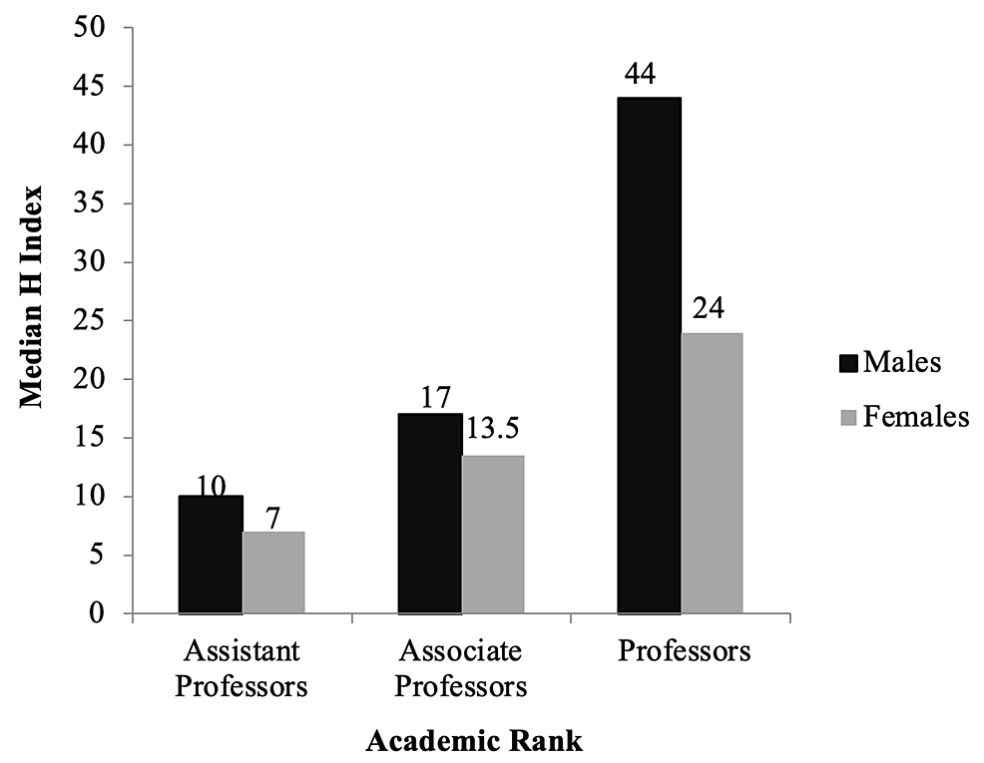
Distribution of median h-index by gender for 140 faculty across academic ranks in medical biochemistry and genetics programs in North America in 2019.

Publication data was available for 144 faculty members. Table 2 lays out the median number of publications, citations, h-index and years of active research for both male and female faculty members. The median number of publications per faculty were 55.5 (1 - 583), with more publications for male than female faculty members across all academic ranks. Citation data were available for 140 faculty members. The median citation per faculty was 1648.5 (0 - 45524), and the median citations of male academic faculty was higher than that of female faculty at all academic ranks. H-index data was available for 140 faculty members (Figure 3). The median h-index was 19.5 (0 - 109), and male academic faculty had a higher h-index at all ranks than female faculty.

**Table 2:** Median values of baseline characteristics for physician biochemistry faculty by gender Values in parenthesis represent ranges

Median values of baseline characteristics for physician biochemistry faculty by gender		
	Males	Females
Number of publications		
Assistant Professors	27 (1-75)	14 (1-27)
Associate Professors	51.5 (1-235)	30 (12-79)
Professors	148 (1-453)	81.5 (2-583)
Number of citations		
Assistant Professors	484 (0-3954)	256 (0-2538)
Associate Professors	1280 (72-10393)	1127 (66-2169)
Professors	7395 (223-45524)	2957.5 (14-42378)
H-index		
Assistant Professors	10 (0-32)	7 (0-14)
Associate Professors	17 (3-49)	13.5 (4-25)
Professors	44 (3-94)	24 (2-109)
Years of active research		
Assistant Professors	13 (2-26)	12 (1-37)
Associate Professors	22 (4-38)	14.5 (8-25)
Professors	31 (12-55)	28 (4-44)

**Figure 4:**
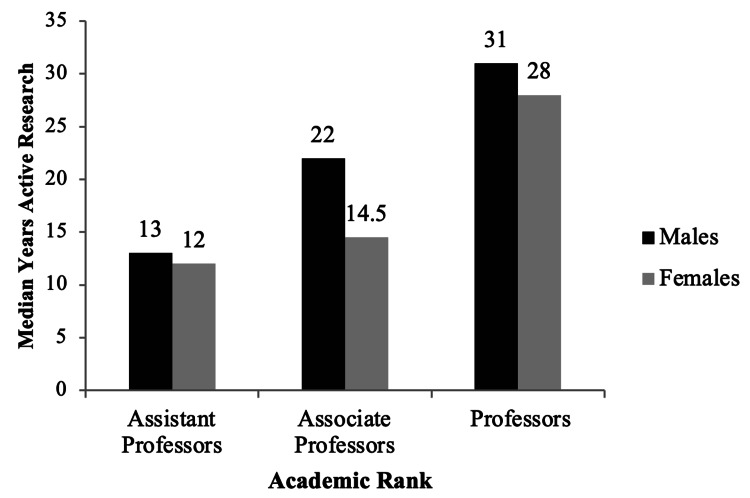
Distribution of median years of active research by gender for 139 faculty members across academic ranks in medical biochemistry and genetics programs in North America in 2019.

Lastly, years of active research data were available for 139 faculty members (Figure 4). The median years of active research per faculty member were 23 (1 - 55), and male faculty had more years of active research than females across all academic ranks.

Upon performing univariate regression analysis, we found that gender, p<0.04), academic rank (r=0.648, p(<0.02), and leadership rank (r=0.491, p<0.02) were significantly related to h-index. Years of active research were insignificant (r=0.451, p<0.71). We found no collinearity between independent variables at a cut-off of 0.8. Our team identified the main effects using a stepwise selection strategy which was based on p<0.05. We dropped leadership rank from the model as it was found to be insignificant. The multivariable analysis supported the inclusion of gender, number of citations, number of publications and academic rank in the preliminary model. We created interaction terms between each of the main effects in the model, and there were no significant interactions. Academic rank and publications were not confounders for h-index; neither were academic rank or number of citations.

The Final Model

y(x) = β0 + β1 (Female) + β2 (Publications) + β3 (citations) + β41 (Academic Rank- Associate Professor) + β42 (Academic Rank- Professor)

H-index was the outcome of interest here. Our research found that the most suitable model comprised gender, median number of publications, median number of citations, and academic rank. This prediction equation accounted for 73% of the variability in the model as adjusted R square = 0.73, F test was 344.87, the p-value was ≤ 0.001. The remaining variability in the model may be explained by variables such as employment status (e.g., full-time versus part-time employment, or contract versus tenure positions), author age or years of employment. However, this was beyond the scope of our paper as we were limited by publicly available data. After running a binary logistic regression, we found that female faculty of medical biochemistry and genetics programs have 0.39 times the odds of having a higher h-index than male faculty, keeping all other variables constant.

## Discussion

Summary of findings

The present study demonstrates greater overall male faculty representation in medical biochemistry and genetics programs across North America, with larger differences present higher up in academic and leadership rank. One explanation for these differences may be poor retention of women between academic ranks due to stress/workload, motherhood, or compensation [13]. This is in line with findings reported by Bennet et al. who found that despite an increase in female basic science faculty in US medical schools, men still represented the majority of basic science faculty overall and at all academic ranks [14]. A global study on gender differences across various disciplines in academia found that women accounted for only 27% of authors with fewer women in mathematics and physics, and more women in fields such as psychology [15].

When it comes to research productivity, male faculty had greater publication output than female faculty across all academic ranks. While differences across genders decreased slightly higher up in academic ranks, women still had just over half of the publications of their male counterparts. Symonds et al. found similar results in the life sciences, with men publishing an average of 40% more papers than women. Interestingly, this study also found that the divergence in publication rates appeared as early as two years after their first publications [16].

Our findings suggest that citations are higher for male faculty than female faculty. This may be due to women having fewer publications than men or differences in citations per article, the latter which is unclear. The Bendels study [17] reported a statistically lower median number of citations per article for key female authors than male authors, while Symonds et al. and Cameron et al. found the opposite [16,18]. Lastly, women faculty showed fewer median years of active research across all academic ranks, with the widest difference being among professors.

Given the higher median number of publications and citations of male faculty, it was unsurprising that the median h-index was greater for men than women across all academic ranks. Furthermore, as h-index is often used to determine promotions, academic rank was statistically associated with the metric. The model also found that when other variables were held constant, women in medical biochemistry, and genetics have 0.39 times the odds of having a higher h-index than men, as has been reported previously [18]. 

Potential barriers for female faculty

There are various factors that may account for the gender differences in career advancement and research productivity of medical biochemistry and genetics faculty. While mentorship has been shown to lead to improvements in the number of female faculty and female department chairs, a lack of access to mentorship opportunities, especially when it comes to addressing the unique needs of female faculty at various career stages, may be a barrier to the career success of women [9]. Women in biomedical careers often spend more time on parenting and domestic responsibilities than men, requiring more family-supportive environments and policies to promote career satisfaction, progression, and retention [19]. Yet institutions may lack such policies or there may be barriers to the utilization of such policies, including inconsistent awareness or implementation, and unsupportive department heads [19, 20]. Promotion is also closely tied to scholarly activities [21]. Since women are more likely to be involved in clinical educator tracks, tied to greater patient care and education, they may be less likely to receive promotions due to traditional promotional requirements [21, 22]. Finally, continued gender bias in academia may hinder women’s productivity and career advancement. Many women in academia continue to report explicit harassment and micro-aggressions [19]. This bias also translates to the institutional level, with a 2016 study of National Institutes of Health grant applications showing that different evaluation standards are applied to men and women when determining grant funding [23]. This bias may impact women’s satisfaction with their careers, as well as their ability to carry out research projects.

Strategies 

Gender disparities can have important negative implications on female faculty job satisfaction, retention, and future advancement [19]. However, there are strategies that can be implemented to address these gender differences and improve the environment for women in medical biochemistry and genetics. A formal mentorship network that includes peer mentors and others with diversity in expertise, rank, and gender, may be implemented in research institutions to accommodate the career stage and mentorship needs of researchers [24]. Furthermore, sponsorship programs facilitating senior members nominating junior members for leadership positions can help to advocate for gender parity in the field [25, 26]. Policies may also be developed to create more family-supportive environments. Changes include access to different child-care options, having written, explicit policies regarding parental leave; flexibility in scheduling and progression through training; and, having more flexibility within the promotion process, such as by stopping the “tenure clock” [20, 27]. Institutions with clinical educator tracks can consider policies around giving equivalence to clinical work and teaching compared to research productivity in the promotional process [22]. Finally, addressing gender bias in academia is a difficult task to tackle. Some steps that can be taken include incorporating explicit, evidence-based anti-sexist training for individuals [28-30].

Study limitations

Some limitations of the study are that there was an inherent degree of potential inaccuracy in obtaining faculty listings from institutional websites, as these may not have been updated in real-time. Moreover, name changes after marriage or divorce could not be accounted for. There was a lack of information regarding faculty with other gender identities. Our study was limited to individuals with MD or DO degrees, and therefore may not be generalizable to all faculty in medical biochemistry and genetics.

## Conclusions

Our results have identified that men are ahead of women in terms of number of publications, citations, academic and leadership rank, years of active research, and h-index. Gender is also independently associated with h-index for medical biochemistry and genetics faculty. Considering new ways to support female scientists may be critical to minimizing the gender productivity gap in medical biochemistry and genetics.
